# Molecular Evidence of Ovine Theileriosis in Selected Areas of Qinghai Province

**DOI:** 10.3390/vetsci13020129

**Published:** 2026-01-29

**Authors:** Lamu Aan, Yi Yang, Peiyao Yang, Chengcai Wang, Zhi Li, Yong Fu, Xueyong Zhang, Dan Jia, Xiuying Shen, Zhihong Guo, Jie Wang, Hong Duo

**Affiliations:** 1 Academy of Animal Sciences and Veterinary Science, Qinghai University, Xining 810016, China; aanlamu@163.com (L.A.); yy18839119285@sohu.com (Y.Y.); ypy1149006182@outlook.com (P.Y.); 18697141556@163.com (C.W.); lizhi19880717@163.com (Z.L.); qhfuyong@163.com (Y.F.); zhang_xyong@163.com (X.Z.); jiadan1995@163.com (D.J.); qhshenxiuying@163.com (X.S.); 1984990033@qhu.edu.cn (Z.G.); 2 Key Laboratory of Animal Disease Pathogen Diagnosis and Green Prevention and Control Technology of Qinghai Province, Qinghai University, Xining 810016, China; 3 Department of Animal Science, Qinghai Agri-Animal Husbandry Vocational College, Xining 812100, China

**Keywords:** ovine theileriosis, Qinghai, *Theileria* spp., genetic diversity, epidemiology, *18S* rRNA

## Abstract

Ovine theileriosis is a tick-borne hemoparasitic disease caused by protozoan parasites of the genus *Theileria*, which poses a substantial threat to sheep and goats. The disease is prevalent in northwestern China, yet studies on the diversity of its pathogens in Qinghai Province remain limited. From 2014 to 2025, 1062 blood samples were collected from sheep in 11 counties of Qinghai Province, and the *Theileria* spp. were investigated by PCR targeting the *18S* rRNA gene. Genetic diversity analysis was performed to assess sequence variations and phylogenetic relationships. The results show that 424 samples tested positive for *Theileria* spp., with an overall infection rate of 39.92%, including *T. uilenbergi* 26.74%, *T. luwenshuni* 22.41%, *T. ovis* 18.17%, *T. capreoli* 0.56%, and *Theileria* sp. OT3 0.09%. The co-infection rate was 22.69%. Six haplotypes were detected in both *T. uilenbergi* and *T. ovis*, and fifteen in *T. luwenshuni*. Hap 1 was the dominant haplotype for all three species, with respective proportions of 73.08%, 77.06% and 82.17%. Phylogenetic analysis showed the strains were most closely related to those from Hunan, Turkey, Shaanxi, etc. These findings reveal a high diversity of *Theileria* species in Qinghai sheep, which provides crucial epidemiological insights into the transmission dynamics of ovine theileriosis in Qinghai and vital support for developing effective control strategies.

## Simple Summary

This study investigates the diversity and epidemiology of *Theileria* species that infect sheep in Qinghai Province, China. Through molecular detection and genetic analysis, a high overall infection rate (39.92%) was found, with three dominant species identified: *Theileria uilenbergi*, *Theileria luwenshuni*, and *Theileria ovis*. Co-infections were frequent (22.69%), reflecting complex transmission patterns in the region. Phylogenetic analysis further revealed that local strains were closely related to those from other areas of China and from Turkey, suggesting possible historical pathogen dispersal. These findings provide key insights into the genetic structure and distribution of *Theileria* parasites in Qinghai, supporting improved surveillance and control strategies for ovine theileriosis.

## 1. Introduction

Ovine theileriosis is a tick-borne hemoprotozoan disease caused by parasites of the genus *Theileria* within the family Theileriidae, which infect vertebrate erythrocytes, macrophages and lymphocytes. This disease causes significant economic losses to the livestock industry [1,2]. To date, more than ten species within the genus *Theileria* have been identified, among which six are known to infect sheep: *Theileria luwenshuni*, *Theileria uilenbergi, Theileria ovis, Theileria lestoquardi, Theileria recondita, and Theileria separata* [3]. Of these, *T. luwenshuni*, *T. uilenbergi*, and *T. lestoquardi* are highly pathogenic and are classified as virulent *Theileria* spp., whereas *T. ovis*, *T. recondita*, and *T. separata* exhibit low pathogenicity and are referred to as benign forms of *Theileria* spp. [4,5,6]. Ovine theileriosis is widely distributed across the world and has been reported in many countries, including Sudan and Algeria in Africa, Spain in Europe, and multiple countries across Asia [7,8,9,10].

To date, the pathogenic species of ovine theileriosis reported in China have included *T. uilenbergi*, *T. luwenshuni*, and *T. ovis* [11,12,13]. Under natural infection conditions, most cases were co-infections involving *T. uilenbergi* and *T. luwenshuni*, with only a minority being single-species infections [14]. In northwestern China, *T. luwenshuni* and *T. uilenbergi* commonly occur in mixed infections, with cases of theileriosis reported in Gansu, Shaanxi, Inner Mongolia, Xinjiang and Qinghai [15,16,17,18,19]. Previous studies have detected *Theileria* species in sheep from certain regions of Qinghai Province, including *T. ovis*, *T. luwenshuni*, *T. uilenbergi*, *Theileria capreoli*, *Theileria* sp. and *Theileria* sp. OT3 [20,21]. However, recent factors such as climate change, international trade and the expansion of the geographical range of ticks may exacerbate the epidemic risk of tick-borne ovine theileriosis in Qinghai Province. Therefore, this study conducted a molecular epidemiological investigation of ovine theileriosis in selected regions of Qinghai Province, aiming to identify the pathogenic species and confirm the genetic diversity of ovine theileriosis. The findings are expected to provide a scientific basis for comprehensive prevention and control of the disease in this area.

## 2. Materials and Methods

### 2.1. Study Area and Sample Collection

**Figure 1:**
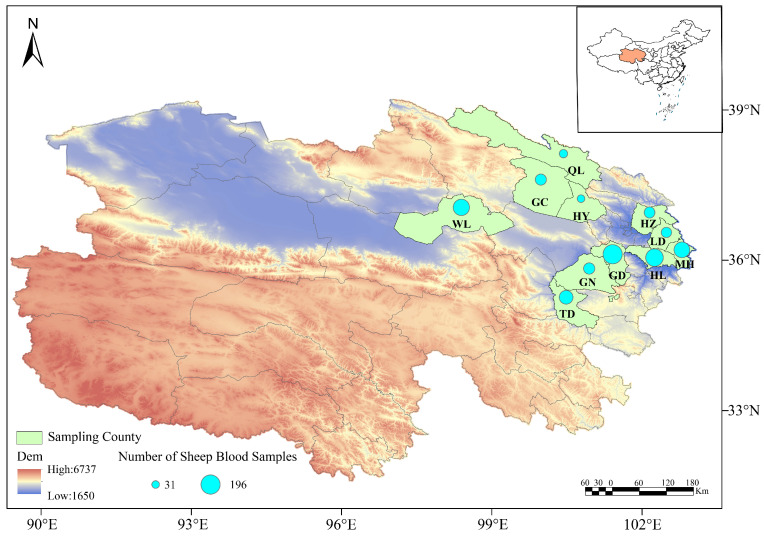
The sampling sites in this study were classified into two major ecological zones: the Eastern Agro-pastoral Ecotone, with an altitude of 2000–3000 m and a plateau continental semi-arid climate, covering Huzhu (HZ), Ledu (LD), Minhe (MH), Hualong (HL) and Guide (GD); and the Circum-Qinghai Lake Pastoral Area, with an altitude of 3000–4000 m and a plateau continental arid climate, covering Qilian (QL), Gangcha (GC), Haiyan (HY), Guinan (GN), Tongde (TD) and Wulan (WL). Differences in altitude and climate between the two zones may affect vector distribution and pathogen transmission dynamics.

A total of 1062 sheep blood samples were collected from 11 counties in Qinghai Province, including Huzhu, Minhe, Hualong, Gangcha, Wulan, Guinan, Guide, Qilian, Haiyan, Ledu, and Tongde during the sampling period from 2014 to 2025. The blood samples were collected using anticoagulant tubes, and stored at 4 °C for subsequent analysis (see Figure 1 and Table 1).

This study employed a geographically stratified sampling strategy, selecting a total of 11 counties within the main ecological-pastoral zones of Qinghai Province as sampling sites: the Eastern Agro-pastoral Ecotone (Huzhu County, Minhe County, Hualong County, Guide County and Ledu District) and the Circum-Qinghai Lake Pastoral Area (Gangcha County, Haiyan County, Qilian County, Guinan County and Tongde County). This scheme systematically covered the major sheep farming systems in the province, ranging from agro-pastoral ecotones to intensive pastoral areas.

This study is a fundamental pathogen detection survey. Field sample collection spanned multiple years, with the main body of work concentrated between 2014 and 2021. Supplementary sampling was conducted in some regions in 2022–2025. All sampling was completed between April and October. The sample sizes (ranging from 31 to 196 per county) were determined based on the sample size formula for detection surveys (*n* ≥ ln(*α*)/ln(1 − *p*), where *α* = 0.05, *p* = 5%), which is fully effective for achieving the core objective of “detecting the presence of pathogens”. In subsequent analyses, prevalence was estimated for counties with sufficient sample sizes, while data from counties with smaller sample sizes were primarily used to confirm the presence of pathogens and for subsequent genetic analysis.

### 2.2. Genomic DNA Extraction

Genomic DNA was extracted from 200 μL of each whole blood sample using the TIANamp Blood DNA Kit (TIANGEN Co., Ltd., Beijing, China) in accordance with the manufacturer’s instructions. The extracted DNA was preserved at −20 °C for subsequent molecular experiments.

### 2.3. PCR Amplification

Four pairs of primers targeting the *18S* rRNA gene of *Theileria* spp. were synthesized on the basis of previous reports, comprising a *Theileria* genus-specific primers set (989s/990as), a *T. luwenshuni*-specific set (Tluw310s/Tluw374as), a *T. uilenbergi*-specific set (Tuil310s/Tuil689as), and a *T. ovis*-specific set (F/R) [16,22,23]. All primers were synthesized by Sangon Biotech (Sangon Biotech Co., Ltd., Shanghai, China) and their detailed sequences and corresponding amplification conditions are shown in Table 2.

PCR amplification was performed on all extracted DNA samples using *Theileria* genus-specific primers (989s/990as). The total reaction volume was 25 μL, containing 12.5 μL of 2× Taq Master Mix (Takara Biomedical Technology Co., Ltd., Dalian, China), 1 μL each of the forward and reverse primers (10 μmol/L), 2 μL of DNA template, and 8.5 μL of RNase-free water (Takara Biomedical Technology Co., Ltd., Dalian, China). Positive and negative controls were included in each PCR reaction. Genomic DNA samples from previously confirmed *Theileria*-positive sheep were used as the positive control, while genomic DNA samples from verified *Theileria*-negative sheep were used as the negative control. The amplification protocol for the *Theileria* genus-specific primers consisted of an initial denaturation at 94 °C for 4 min, followed by 35 cycles of denaturation at 94 °C for 30 s, annealing at 55 °C for 1 min, and extension at 72 °C for 1 min 10 s, with a final extension at 72 °C for 10 min.

The amplified PCR products were analyzed by 1.2% agarose gel electrophoresis. Amplified fragments of the expected size were purified, and the gel-extracted products were sent to Sanger technology (Sangon Biotech Co., Ltd., Shanghai, China) for sequencing.

All 424 PCR-positive samples were subjected to Sanger sequencing without exclusion. To ensure the reliability of the final sequence data, particularly for samples that showed faint or non-specific bands in the initial screening, we implemented a strict quality control protocol prior to sequencing as follows:

(1) Re-amplification with Optimized Conditions: For these samples, PCR conditions (e.g., annealing temperature and extension time) were re-optimized and the reactions were repeated to enhance the yield and specificity of the target fragment.
(2) Gel Separation and Purification: The re-amplified products were separated on a 1.5% agarose gel. The specific target band was precisely excised and purified using a commercial gel extraction kit (TIANGEN Co., Ltd., Beijing, China) to remove impurities such as primer dimers and non-specific products.
(3) Purity Verification and Quantification: The purified products were verified using a NanoDrop spectrophotometer. Samples proceeded to sequencing only when the A260/A280 ratio fell within the optimal range of 1.8–2.0, confirming high purity.

The samples that tested positive with the *Theileria* genus-specific primers were subsequently subjected to species-specific PCR amplification using primers targeting *T. luwenshuni*, *T. uilenbergi*, and *T. ovis*, respectively. All PCR products were analyzed by 1.5% agarose gel electrophoresis. The obtained nucleotide sequences of *Theileria* spp. were aligned using the BLAST program (https://blast.ncbi.nlm.nih.gov/Blast.cgi, accessed on 8 October 2025) within the NCBI database to confirm both the identity of the target gene and the *Theileria* spp.

### 2.4. Genetic Diversity Analyses

All sequences used for downstream analyses were derived from PCR products amplified using the *Theileria* genus-specific primers. PCR products from species-specific primers were not sequenced, as their specificity was confirmed via control experiments. The sequence variants used for haplotype analysis in this study were all derived from diploid nuclear genes (*18S* rRNA). The genetic diversity of the obtained ovine *Theileria* spp. *18S* rRNA gene sequences was analyzed using DnaSP v6 software, including the number of haplotypes (H), the number of polymorphic sites (S), haplotype diversity (Hd) with its standard deviation (SD), and nucleotide diversity (π) with its standard deviation (SD). Multiple sequence alignment was performed using the MAFFT program implemented in PhyloSuite v1.2.3, followed by the trimming of the aligned sequences with TrimAI to remove low-quality and ambiguous regions, thereby enhancing the reliability of the phylogenetic tree. The validated gene sequences were subjected to sequence comparison and phylogenetic analysis. A phylogenetic tree of the *Theileria* spp. *18S* rRNA gene was subsequently constructed with IQ-TREE v2.2.0 using the Maximum Likelihood method and *Babesia microti* (accession no. AB242176) was used as the outgroup.

### 2.5. Statistical Analyses

To assess the geographical significance of differences in the prevalence of *Theileria* spp. across 11 counties, statistical analyses were performed in this study. The 95% confidence intervals (95% CIs) for the prevalence rates were calculated using VassarStats (VassarStats: Statistical Computation Web Site, https://vassarstats.net/, accessed on 6 January 2026) to estimate the potential range of the true prevalence. An overall heterogeneity test was conducted on the prevalence rates of all counties using the chi-square test. After confirming the existence of a significant overall difference, pairwise comparison tests were further performed for all county combinations. The significance level of the tests was adjusted using the Bonferroni correction method.

## 3. Results

### 3.1. Pathogen Diversity of Ovine Theileria

**Figure 2:**
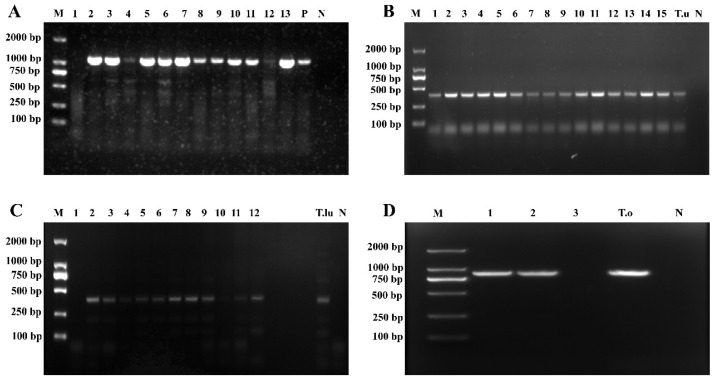
Electrophoretogram of *Theileria* spp. *18S* rRNA gene amplification. (**A**) Results of *Theileria* genus-specific primers amplification; (**B**) Results of *T. uilenbergi*-specific primer amplification; (**C**) Results of *T. luwenshuni*-specific primer amplification; (**D**) Results of *T. ovis*-specific primer amplification (M: Marker; 1–15: Test samples; N: Negative control; P: Positive control; T.u: *T. uilenbergi* samples; T.lu: *T. luwenshuni* samples; T.o: *T. ovis* samples).

Based on the results of PCR amplification using *Theileria* genus-specific primers, 424 out of the 1062 whole-sheep-blood genomic DNA samples yielded a target band of approximately 1033 bp (Figure 2A), indicating the presence of *Theileria* spp. infection in these samples. A total of 424 positive samples were detected across the 11 counties in Qinghai Province, yielding an overall infection rate of 39.92% (424/1062), including Hualong County (77.44%, 127/164), Ledu District (76.79%, 43/56), Huzhu County (70.15%, 47/67), Guide County (63.78%, 125/196), Minhe County (47.73%, 63/132), Qilian County (13.51%, 5/37), Haiyan County (9.68%, 3/31), Tongde County (8.00%, 8/100), and Wulan County (2.08%, 3/144). Notably, no infection was detected in two counties: Guinan and Gangcha (Table 3).

A total of 230 sequences of *T. luwenshuni*, 78 sequences of *T. uilenbergi*, 109 sequences of *T. ovis*, 6 sequences of *T. capreoli*, and 1 sequence of *Theileria* sp. OT3 were successfully amplified, sequenced, and verified from the collected sheep blood samples. Only the distinct haplotype sequences identified in this study have been submitted to the NCBI GenBank: *T. luwenshuni* [PX700466-PX700480], *T. uilenbergi* [PX694670-PX694675], *T. ovis* [PX830760-PX830765], *T*. *capreoli* [PX830757-PX830758], and *Theileria* sp. OT3 [PX830759]. The sequencing results reveal nucleotide sequence identities of 99.62–99.90% for *T. uilenbergi* (MW881299), 99.18–100% for *T. luwenshuni* (OR104981), and 99.20–99.88% for *T. ovis* (MW020235). One sequence showed a 97.64% identity with *Theileria* sp. OT3 (MG930118) from GenBank and *Theileria* sp. OT3 274 (GD) sequence was most closely related to *Theileria* sp. OT3 MG930117. Six sequences exhibited 98.16–99.90% identity with *T. capreoli* (KJ188219) in GenBank and were identified as *T. capreoli*.

The 424 samples that tested positive with *Theileria* genus-specific primers were further amplified using species-specific primers for *T. luwenshuni*, *T. uilenbergi*, and *T. ovis*. Among these, amplification with *T. uilenbergi*-specific primers yielded an approximately 388 bp target band in 284 samples (Figure 2B), indicating that the overall infection rate of *T. uilenbergi* was 26.74% (284/1062), including Hualong County (71.95%, 118/164), Guide County (52.55%, 103/196), Ledu District (48.21%, 27/56), Huzhu County (19.40%, 13/67), and Minhe County (17.42%, 23/132).

The amplification with *T. luwenshuni*-specific primers yielded an approximately 389 bp target band in 238 samples (Figure 2C), indicating that the overall infection rate of *T. luwenshuni* was 22.41% (238/1062), including Ledu District (57.14%, 32/56), Huzhu County (52.24%, 35/67), Hualong County (47.56%, 78/164), Minhe County (28.79%, 38/132), and Guide County (28.06%, 55/196).

Amplification with *T. ovis*-specific primers produced an approximately 904 bp target band in 193 samples (Figure 2D), confirming *T. ovis* infection in these samples. The overall infection rate was 18.17% (193/1062), including Hualong County (64.63%,106/164), Guide County (28.06%, 55/196), Ledu District (23.21%, 13/56), Qilian County (13.51%, 5/37), Haiyan County (9.68%, 3/31), Tongde County (8.00%, 8/100), and Wulan County (2.08%, 3/144).

Of the 424 samples successfully amplified with *Theileria* genus-specific primers, 6 samples failed to yield amplicons in the species-specific primer PCR assay. These 6 samples were subsequently confirmed as *T. capreoli* on the basis of amplicons from the *Theileria* genus-specific primer PCR, combined with Sanger sequencing and phylogenetic analysis.

**Figure 3:**
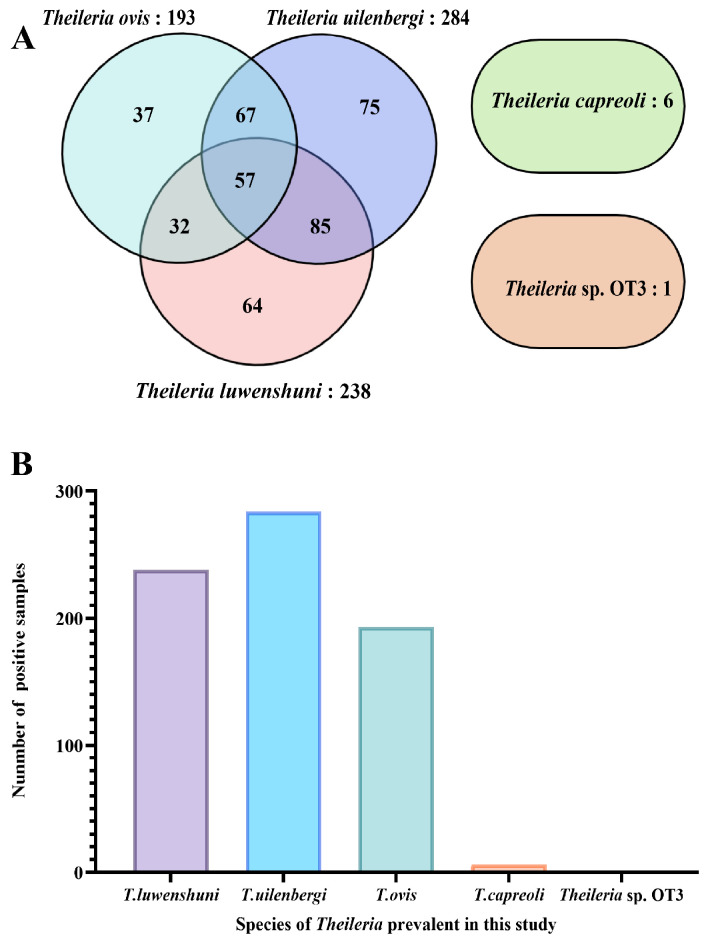
Infection profile of *Theileria* species based on *18S* rRNA gene PCR amplification. (**A**) Venn diagram depicting co-infection patterns among different *Theileria* species. Each ellipse represents a species (*T. uilenbergi*: 284 samples, purple; *T. luwenshuni*: 238 samples, pink; *T. ovis*: 193 samples, blue; *T. capreoli*: 6 samples, green). Numbers in non-overlapping areas indicate single-species infections, while overlapping areas represent co-infections (e.g., 85 samples co-infected with *T. uilenbergi* and *T. luwenshuni*). The separate ellipse on the right represents *Theileria* sp. OT3 (only 1 single-infection sample, orange). (**B**) Bar chart showing the number of positive samples for each *Theileria* species detected in this study: *T. luwenshuni* (238), *T. uilenbergi* (284), *T. ovis* (193), *T. capreoli* (6), and *Theileria* sp. OT3 (1).

Of the 1062 samples, 241 were positive for mixed infections, yielding an infection rate of 22.69% (241/1062), while 183 samples showed single-species infection, with the infection rate of 17.23% (183/1062). Among the single-species infections, the cases were distributed as follows: *T. luwenshuni* (64, 6.03%), *T. uilenbergi* (75, 7.06%), *T. ovis* (37, 3.48%), *T. capreoli* (6, 0.56%), and *Theileria* sp. OT3 (1, 0.09%) (Figure 3).

### 3.2. Polymorphism Analysis and Haplotype Network Construction

The 78 validated *18S* rRNA sequences of *T. uilenbergi* confirmed by NCBI BLAST were analyzed with DnaSP v6, and the results revealed six haplotypes (H), with an Hd of 0.456, a π of 0.00683, and 42 polymorphic sites (S). For the 109 *18S* rRNA sequences of *T. ovis*, six haplotypes were identified, with an Hd of 0.506, a π of 0.00761, and an S of 23. Among the 230 *18S* rRNA sequences of *T. luwenshuni*, 15 haplotypes were observed, with an Hd of 0.323, a π of 0.00504, and an S of 64 (Table 4).

**Figure 4:**
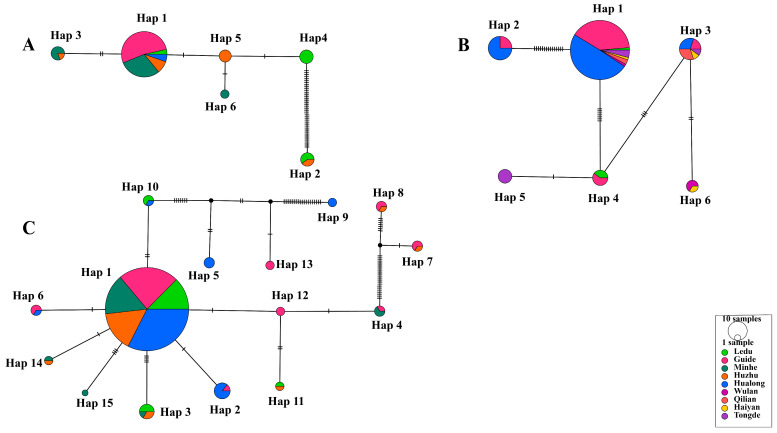
Infection profile of *Theileria* species based on *18S* rRNA gene PCR amplification. (**A**) *T. uilenbergi* (78 sequences), (**B**) *T. ovis* (109 sequences), and (**C**) *T. luwenshuni* (230 sequences). All networks were generated using the TCS method. Circle area is proportional to haplotype frequency (with “10 samples” in the legend as a frequency reference). Different colors represent sampling locations (9 locations total). Connecting lines indicate single-nucleotide mutations, with black ticks denoting mutation steps (absence of ticks indicates one mutation). Small uncolored circles represent hypothetical intermediate haplotypes. Haplotypes are numbered in descending order of their frequency within each species.

Among the six haplotypes of *T. uilenbergi*, Hap 1 (57/78) was predominant and distributed across multiple counties including Guide, Minhe, Huzhu and Hualong, and Ledu District (Figure 4A). Similarly, the predominant Hap 1 (84/109) of *T. ovis* was found across a wide geographical range spanning seven counties (Figure 4B). For *T. luwenshuni*, the most prevalent Hap 1 (189/230) was also detected in five major endemic counties (Figure 4C). The widespread distribution of these predominant haplotypes across multiple, non-contiguous counties may suggest frequent historical gene flow or recent pathogen expansion within the surveyed region. However, no strict phylogeographical structure (i.e., exclusive haplotype region associations) was observed for these species, indicating a lack of strong geographical isolation at the scale of this study.

### 3.3. Phylogenetic Analysis

**Figure 5:**
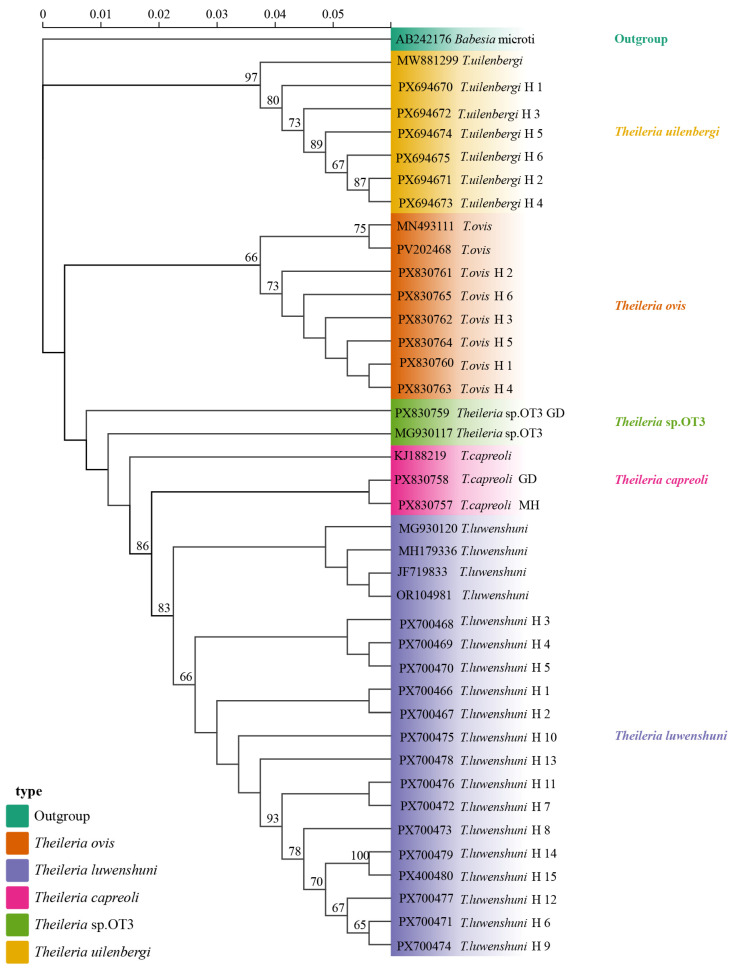
The phylogenetic tree was constructed using the Maximum Likelihood method in IQ-TREE v2.2.0 with *Babesia microti* (AB242176) as the outgroup (highlighted in green). Branches are color-coded by species: *T. uilenbergi* (yellow), *T. ovis* (orange), *T. luwenshuni* (light purple), *T. capreoli* (pink), and *Theileria* sp. OT3 (green). Haplotypes H1–H6 (*T. uilenbergi*), H1–H6 (*T. ovis*), H1–H15 (*T. luwenshuni*), along with *T. capreoli* GD, *T. capreoli* MH, and *Theileria* sp. OT3 represents isolates obtained in this study. Branch labels include sequence accession numbers and haplotype identifiers. The scale bar indicates a genetic distance of 0.05.

Phylogenetic analysis based on the *18S* rRNA gene sequences revealed that the *T. uilenbergi* sequences obtained from sheep blood samples in Qinghai clustered with MW881299 (Hunan). The *T. ovis* sequences formed a clade with MN493111 (Turkey) and PV202468 (Hubei). *T. luwenshuni* sequences clustered with OR104981 (Qinghai), MG930120 (Shaanxi), JF719833 (Gansu), and MH179336 (Gansu). Meanwhile, the *T. capreoli* (MH and GD) sequences grouped with KJ188219 (China), and the *Theileria* sp. OT3 (GD) sequence clustered with *Theileria* sp. OT3 MG930117 (Shaanxi) (Figure 5).

### 3.4. Statistical Results of Geographical Differences in Theileria *spp.* Prevalence

The overall prevalence rate in this survey was 39.92% (424/1062). There were significant geographic differences in prevalence, with rates across counties ranging from 0% to 77.44%. Statistical grouping based on pairwise comparison tests (Table 5) indicated that Hualong County, with the highest prevalence rate, was classified into Group a. Ledu and Huzhu counties were both categorized into Group ab, Guide County into Group b, and Minhe County into Group c, reflecting a general descending trend in prevalence. Notably, Qilian, Haiyan, Tongde, Wulan, Guinan, and Gangcha counties exhibited relatively low infection rates (0–13.51%) and were assigned to Groups d through f (specifically d, d, de, ef, f, f), collectively forming a low-infection region.

## 4. Discussion

In the present study, the epidemiological investigation of ovine theileriosis in selected areas of Qinghai Province revealed an infection rate of 39.92%, which is significantly lower than that in neighboring regions such as Gansu Province and Xinjiang, indicating that Qinghai is a low-prevalence area for ovine theileriosis [24,25]. However, this study found infection rates of *Theileria* spp. exceeding 70% in Ledu District, Huzhu County, and Hualong County, and in Guide County, the infection rate of the pathogen was more than 60%. In contrast, the prevalence of *Theileria* spp. was less than 10.00% in Wulan County, Haiyan County, and Tongde County, while no infection was detected in Gangcha County or Guinan County. These marked disparities suggest a potential link to significant geographical and climatic variations (altitude and temperature) across these regions (Figure 1), which may contribute to the reduced infection rates of ovine theileriosis. The pattern could also be associated with ecological differences in the geographical distribution of vector ticks. These findings are consistent with the previous reports, which demonstrated a close correlation between the distribution of *Haemaphysalis qinghaiensis* and the transmission intensity of *Theileria* spp. in Qinghai [26].

In this study, the infection rates of *T. uilenbergi* and *T. luwenshuni* were 26.74% (284/1062) and 22.41% (238/1062), respectively. These species were predominantly distributed in Guide, Hualong, Minhe, Huzhu, and Ledu, indicating *T. uilenbergi* and *T. luwenshuni* as the dominant species in these regions. In contrast, the infection rate of *T. ovis* in the surveyed areas of Qinghai was 18.17% (193/1062). Previous studies have indicated that *T. ovis* is primarily endemic to the Xinjiang region [25]. Its presence in Qinghai may be attributed to the province’s geographical proximity to Xinjiang and factors such as inter-regional animal movement and expansion of ticks, which could have facilitated the introduction of this pathogen into Qinghai. In addition, this study documented a case of sheep infected with *Theileria* sp. OT3 in the Guide area, whereas the first identification of this pathogen in China was reported in Xinjiang [27]. The natural hosts of *T. capreoli* were initially confirmed to be roe deer and other wild cervids, and previous studies have reported the detection of cross-species infection by this pathogen in sheep in the Qinghai–Tibetan Plateau Area [28,29]. In this study, *T. capreoli* was detected in sheep with an infection rate of 0.56% (6/1062), further confirming its cross-species transmission potential and providing new empirical evidence for the expansion of this pathogen’s host range. This phenomenon is hypothesized to be closely associated with wildlife, domestic animals, and vector ticks sharing habitats, where niche overlap may facilitate the cross-species transmission of the pathogen. In Qinghai Province, *T. capreoli* infections in sheep were identified in regions where *Haemaphysalis qinghaiensis* is the predominant tick species, suggesting that *H. qinghaiensis* may also serve as a potential vector for *T. capreoli* [26]. Concurrently, mixed infections involving two or three *Theileria* species were observed, with a co-infection rate of 22.69%, which highlights the complex nature of pathogen composition of ovine theileriosis in Qinghai Province, which in turn poses significant challenges for its control and prevention. The significant spatial heterogeneity in infection rates may be associated with inherent differences in the ecological/production systems across the study regions. For example, the longer period of indoor or confined rearing in the Eastern Agro-pastoral Ecotone may limit vector contact, whereas the extensive seasonal grazing practices in the Circum—Qinghai Lake Pastoral Area could increase exposure risks. Although this study did not directly measure micro-environmental variables, the ecological zoning used as the basis for sampling inherently integrates key background differences such as altitude and climate (see Section 2.1). These factors warrant validation as quantitative risk factors in future research.

The genetic diversity analysis of the *Theileria*-positive samples revealed haplotype diversity (Hd) values of 0.456 for *T. uilenbergi*, 0.506 for *T. ovis*, and 0.323 for *T. luwenshuni*. These low Hd values indicate a relatively limited level of genetic diversity for these three *Theileria* species in Qinghai, suggesting the existence of relatively stable parasite populations in the region.

Haplotype network analysis revealed that Hap 1 (57/78) was the predominant haplotype of *T. uilenbergi,* distributed across Guide, Minhe, Huzhu and Hualong counties, and Ledu District (Figure 4A). For *T. ovis*, Hap 1 (84/109) was predominant and was found in Hualong, Guide, Tongde, Qilian, Ledu, Wulan, and Haiyan counties (Figure 4B). For *T. luwenshuni*, Hap 1 (189/230) was identified as the predominant haplotype, showing a wide distribution across Hualong, Guide, Minhe, and Huzhu counties, as well as Ledu District (Figure 4C). Notably, the number of haplotypes detected in specific counties varied significantly: Guide County recorded three *T. ovis* and eight *T. luwenshuni* haplotypes, while Hualong County recorded four and seven, respectively. Furthermore, both Ledu District and Minhe County registered three *T*. *uilenbergi* haplotypes each. In summary, Guide and Hualong Counties may represent genetic differentiation centers for *T*. *ovis* and *T. luwenshuni* in Qinghai, while Ledu District and Minhe County are likely focal points for the genetic differentiation of *T. uilenbergi*. This distribution pattern is probably shaped by the combined influences of environmental characteristics of natural foci, host population densities and spatial variations in the distribution of ticks.

This study’s sampling design (purposive sampling with unequal sample sizes across counties) may influence the precision of prevalence estimates and their comparability across counties, and was primarily constrained by resources and sampling accessibility. However, this is aligned with the study’s positioning as a fundamental pathogen detection survey, and the existing sample sizes are sufficient to achieve the core objectives of confirming pathogen distribution and obtaining genetic samples. The widespread distribution of these predominant haplotypes across multiple, non-contiguous counties may suggest frequent historical gene flow or recent pathogen expansion within the surveyed region. However, no strict phylogeographical structure (i.e., exclusive haplotype–region associations) was observed for these species, indicating a lack of strong geographical isolation at the scale of this study.

To further elucidate evolutionary mechanisms and transmission risks, further studies should focus on investigating the population’s genetic structure and the diversity of ticks across extensive regions. This will help systematically clarify the co-evolutionary relationships among ticks, hosts and *Theileria* pathogens, thereby providing a scientific basis for risk assessment and the development of control strategies against tick-borne theileriosis in Qinghai.

## 5. Conclusions

This study confirms that *Theileria* infections in sheep are widespread yet exhibit significant regional variations across the agro-pastoral areas of Qinghai Province. The pathogen displays distinct genetic evolutionary characteristics, with most strains clustering closely with those from neighboring regions, indicating that geographical distance influences its genetic differentiation and transmission. The parasite population demonstrates moderate genetic diversity, and a few dominant genotypes are widely distributed. Variations in infection rates are closely associated with geographical environment, climatic conditions, and breeding scale. These findings clarify the epidemiological patterns and genetic features of *Theileria* in the region, providing a critical basis for implementing targeted regional prevention and control measures.

## Figures and Tables

**Table 1:** Distribution of detected ovine *Theileria* samples by sampling region and time period.

Sampling Sites	Sampling Time	Number of Tested Samples
Huzhu	2014–2016	67
Minhe	2018, 2020–2021	132
Hualong	2020–2021	164
Gangcha	2022	67
Wulan	2022	144
Guinan	2022	68
Guide	2024	196
Qilian	2019	37
Haiyan	2020	31
Ledu	2017	56
Tongde	2025	100
Total		1062

**Table 2:** PCR primers and amplification conditions for ovine *Theileria*.

Target Pathogen	Sequence 5′−3′	Product Length (bp)	Annealing Temperature
*Theileria* spp.	989s: AGTTTCTGACCTATCAG 990as: TTGCCTTAAACTTCCTTG	1033	55 °C
*T. luwenshuni*	Tluw310s: GGTAGGGTATTGGCCTACTGA Tluw374as: TCATCCGGATAATACAAGT	389	57 °C
*T. uilenbergi*	Tuil310s: GGTAGGGTATTGGCCTACCGG Tuil689as: ACACTCGGAAAATGCAAGCA	388	55 °C
*T. ovis*	F: TTTTGCTCCTTTACGAGTCTTT R: TCGTTCACGATTAATAATTGCA	904	55 °C

**Table 3:** Infection status of ovine *Theileria*.

Sampling Sites	Number of Tested Samples	Number of Positive Samples	Infection Rate (%)	*Theileria* spp.	Co- Infection
Huzhu	67	47	70.15	*T. uilenbergi*: 13 *T. luwenshuni*: 35	1
Minhe	132	63	47.73	*T. uilenbergi*: 23 *T. luwenshuni*: 38 *T. capreoli*: 5	3
Hualong	164	127	77.44	*T. uilenbergi*: 118 *T. luwenshuni*: 78 *T. ovis*: 106	120
Gangcha	67	0	0	0	0
Wulan	144	3	2.08	*T. ovis*: 3	0
Guinan	68	0	0	0	0
Guide	196	125	63.78	*T. uilenbergi*: 103 *T. luwenshuni*: 55 *T. ovis*: 55 *T. capreoli*: 1 *Theileria* sp. OT3: 1	88
Qilian	37	5	13.51	*T. ovis*: 5	0
Haiyan	31	3	9.68	*T. ovis*: 3	0
Ledu	56	43	76.79	*T. uilenbergi:* 27 *T. luwenshuni:* 32 *T. ovis*: 13	29
Tongde	100	8	8.00	*T. ovis*: 8	0
Total	1062	424	39.92	*T. uilenbergi*: 284 *T. luwenshuni*: 238 *T. ovis:* 193 *T. capreoli*: 6 *Theileria* sp. OT3: 1	241

**Table 4:** Genetic diversity analysis of ovine *Theileria* sequences.

*Theileria* spp.	Number of Sequences	Number of Haplotypes	Numbers of Polymorphic Sites	Haplotype Diversity (Mean ± SD)	Nucleotide Diversity (Mean ± SD)
*T. uilenbergi*	78	6	42	0.456 ± 0.068	0.00683 ± 0.00331
*T. luwenshuni*	230	15	64	0.323 ± 0.041	0.00504 ± 0.00266
*T. ovis*	109	6	23	0.506 ± 0.054	0.00761 ± 0.00367

**Table 5:** Prevalence and statistical significance grouping across counties in Qinghai Province.

County	Positives	Total	Prevalence (%)	95% CI
Hualong	127	164	77.44 ^a^	70.46–83.17
Ledu	43	56	76.79 ^ab^	64.24–85.90
Huzhu	47	67	70.15 ^ab^	58.35–79.77
Guide	125	196	63.78 ^b^	56.85–70.18
Minhe	63	132	47.73 ^c^	39.39–56.19
Qilian	5	37	13.51 ^d^	5.08–29.57
Haiyan	3	31	9.68 ^d^	2.53–26.90
Tongde	8	100	8.00 ^de^	4.11–15.00
Wulan	3	144	2.08 ^ef^	0.71–5.94
Guinan	0	68	0.00 ^f^	0–6.66
Gangcha	0	67	0.00 ^f^	0–6.76
Total	424	1062	39.92	37.07–42.90

Counties sharing the same superscript letter do not differ significantly in prevalence. The order of the letters denotes group membership only and does not imply any rank order.

## Data Availability

The data presented in this study are openly available in the NCBI GenBank database, where the 18S rRNA sequence data generated in this study have been deposited under accession numbers PX700466 to PX700480 (*T. luwenshuni*), PX694670 to PX694675 (*T. uilenbergi*), PX830760 to PX830765 (*T. ovis*), PX830757 to PX830758 (*T. capreoli*), and PX830759 (*Theileria* sp. OT3).

## Abbreviations

The following abbreviations are used in this manuscript:

*T*. *luwenshuni*	*Theileria luwenshuni*
*T*. *uilenbergi*	*Theileria uilenbergi*
*T*. *ovis*	*Theileria ovis*
*Theileria* spp.	*Theileria* species
*T. capreoli*	*Theileria**capreoli*
H, Hap	haplotypes
S	number of polymorphic sites
Hd	haplotype diversity
π	nucleotide diversity
